# Supplementary material for: Transcriptional Profiling Reveals Mesenchymal Subtypes of Small Cell Lung Cancer with Activation of the Epithelial-to-Mesenchymal Transition and Worse Clinical Outcomes
Source: Cancers (Basel). 2022 Nov 15;14(22):5600. doi: 10.3390/cancers14225600 (PMC9688413; doi:10.3390/cancers14225600)
Supplement: Supplementary file 1 [file cancers-14-05600-s001.zip › SupplementaryFigures_ver4.pdf]

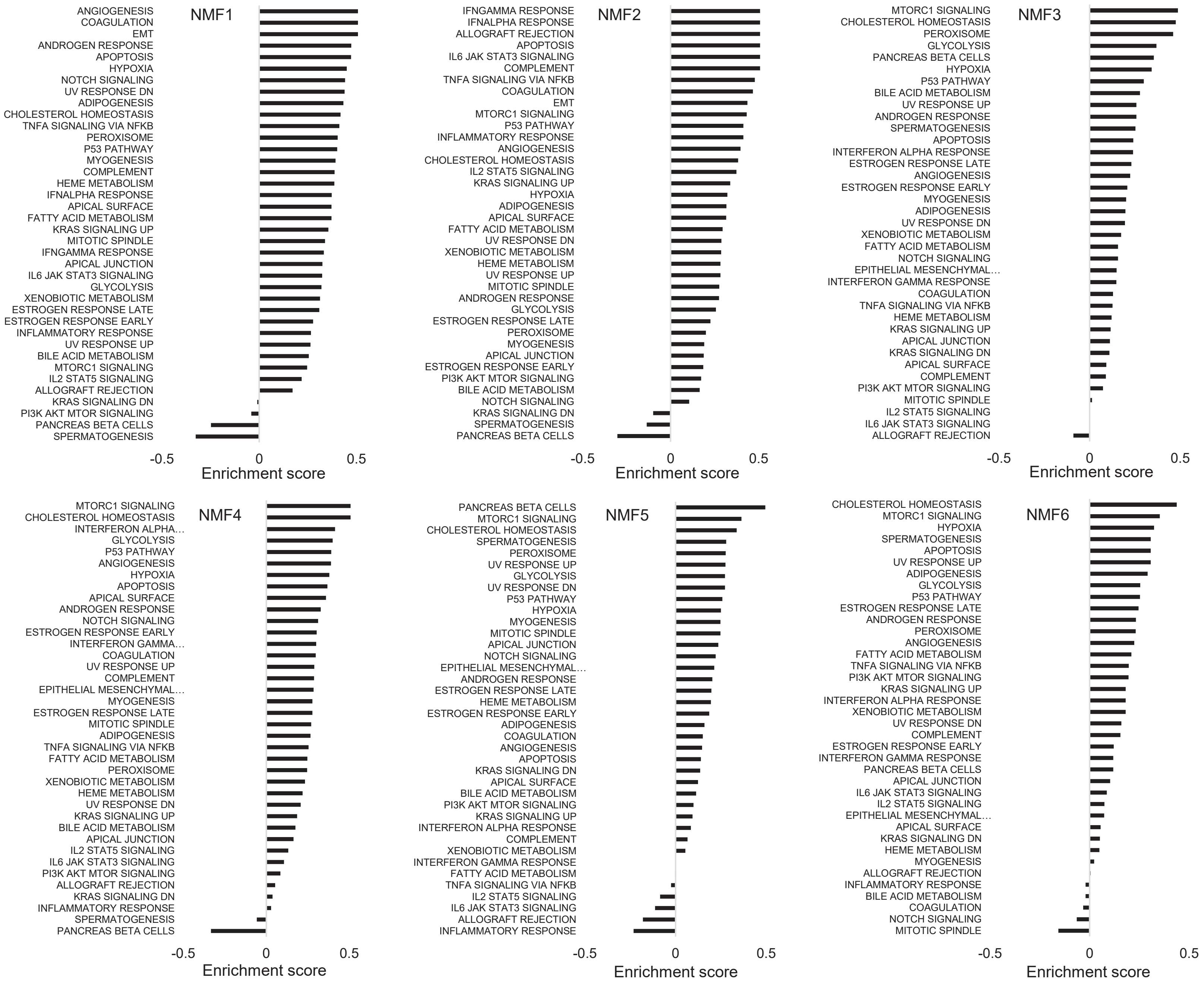

Supplementary Figure S1

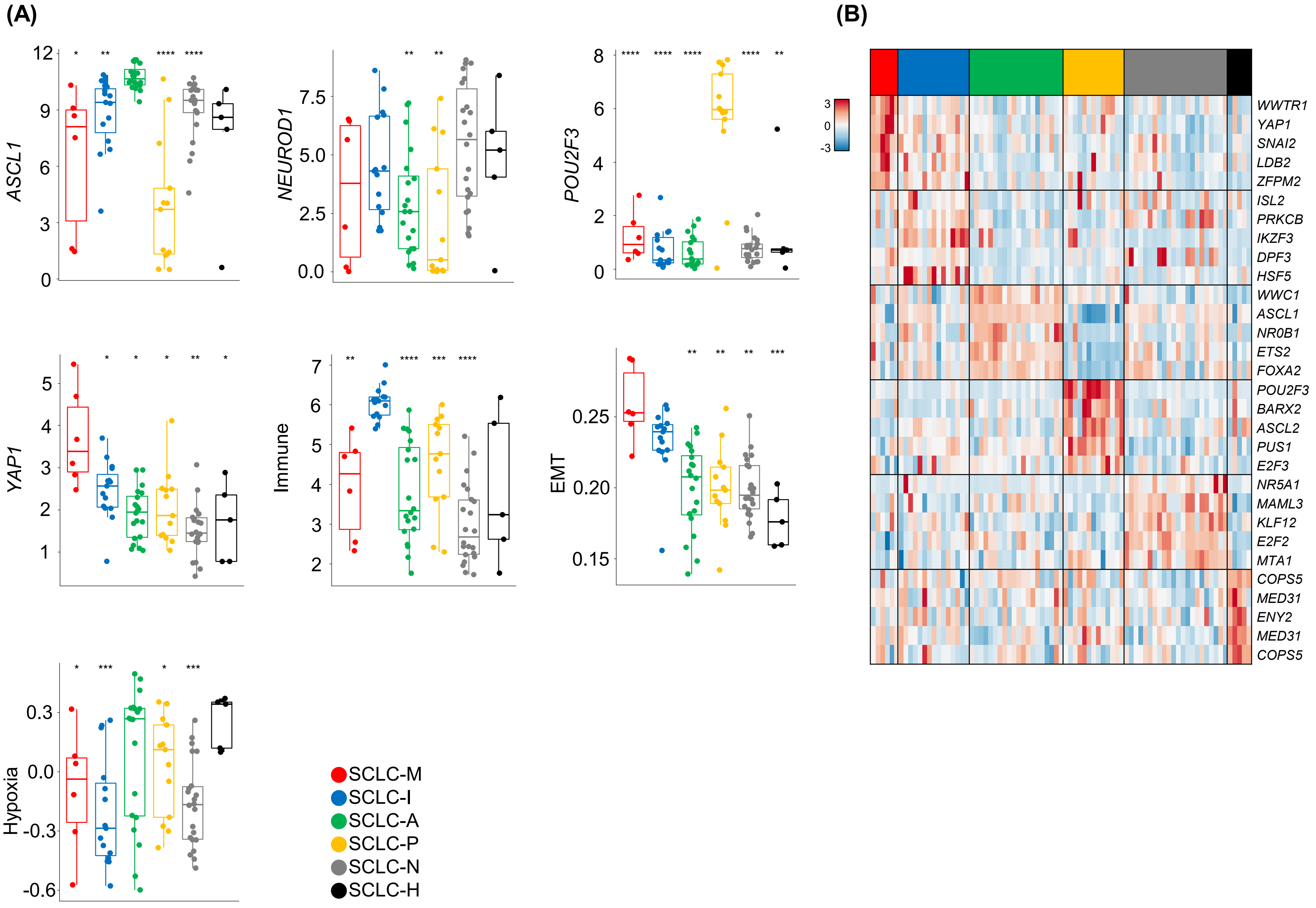

Supplementary Figure S2

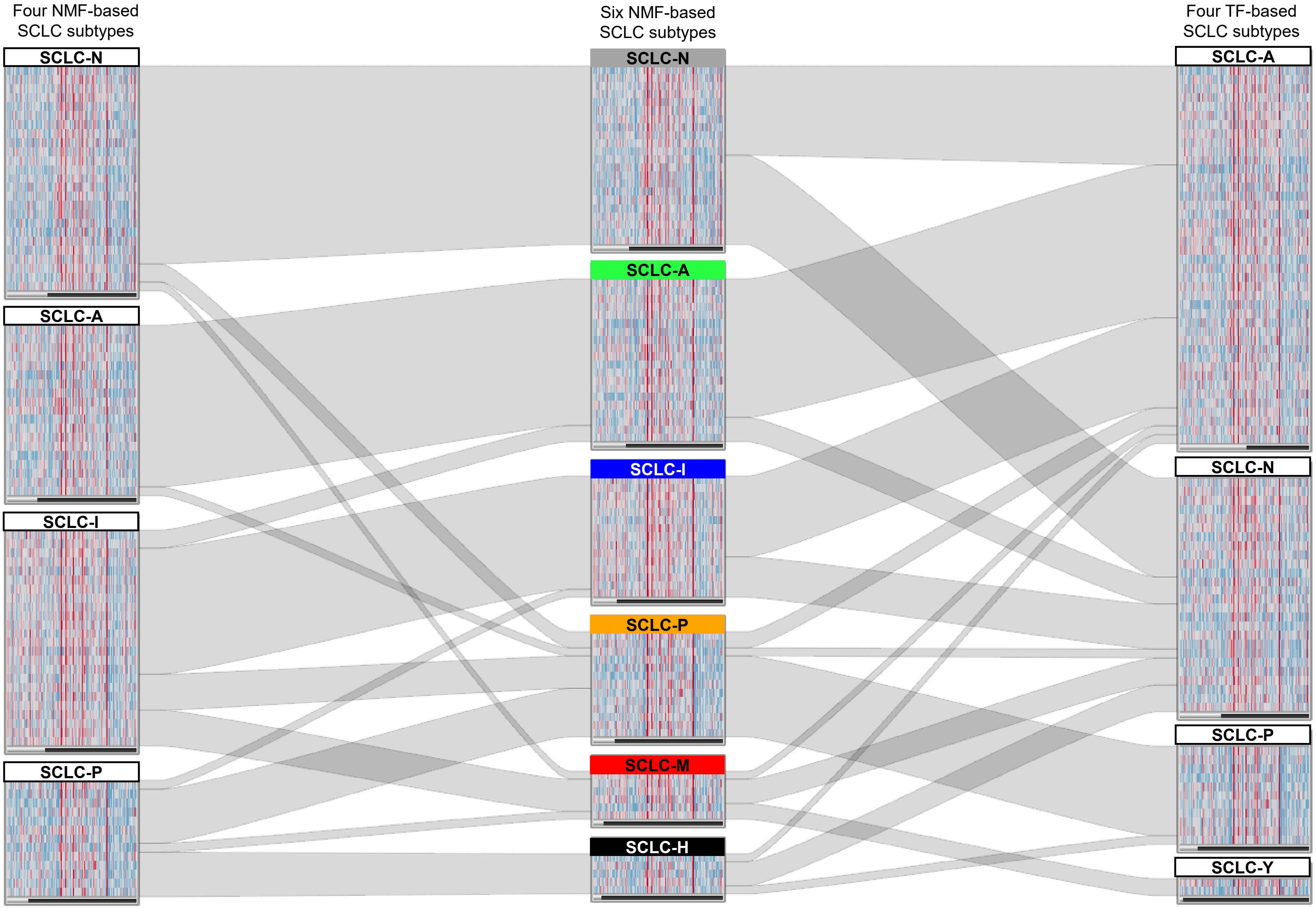

Supplementary Figure S3

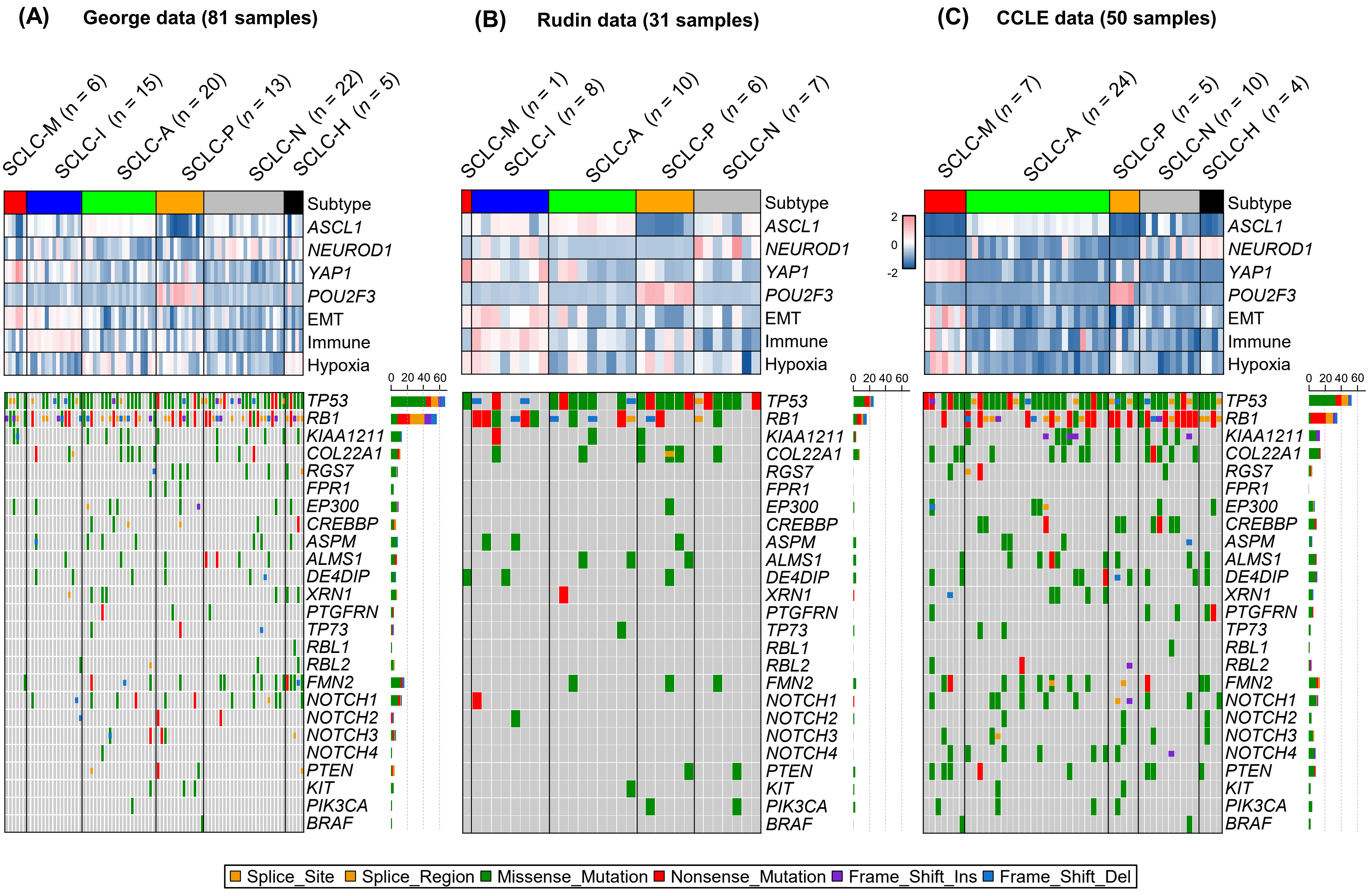

Supplementary Figure S4

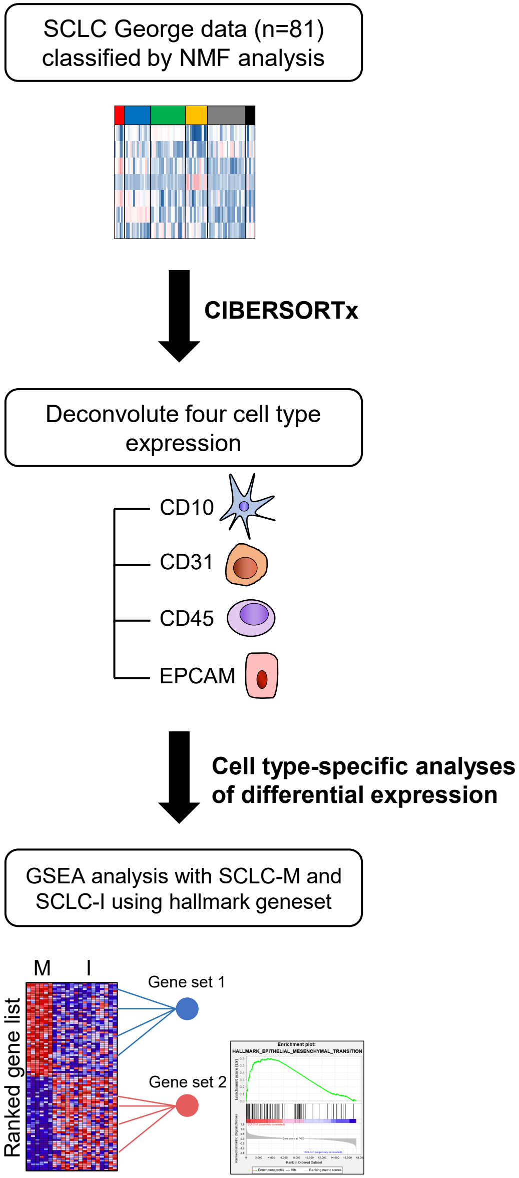

Supplementary Figure S5

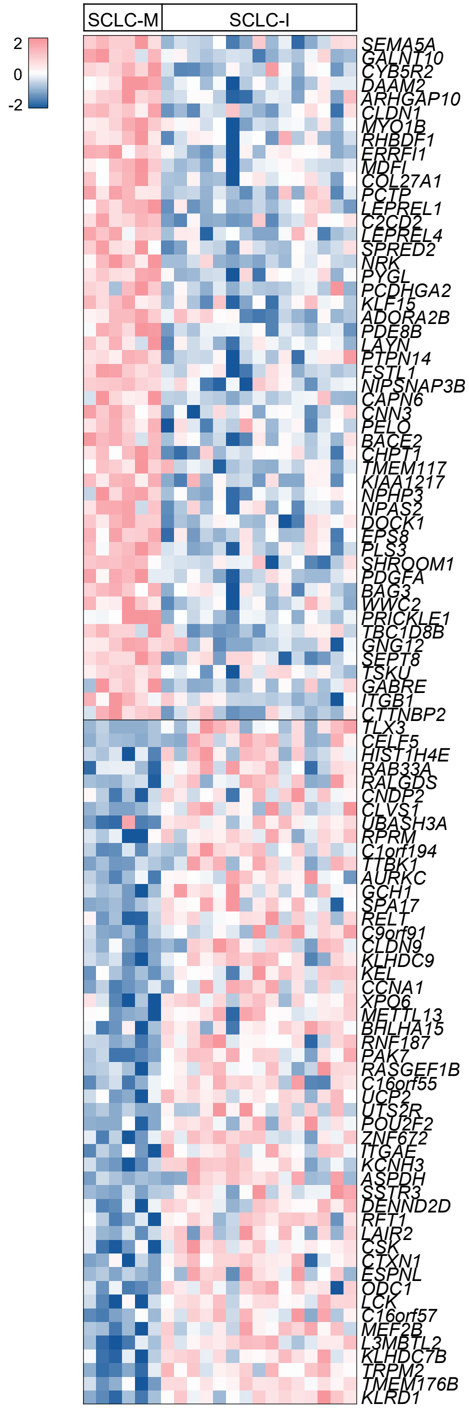

Supplementary Figure S6

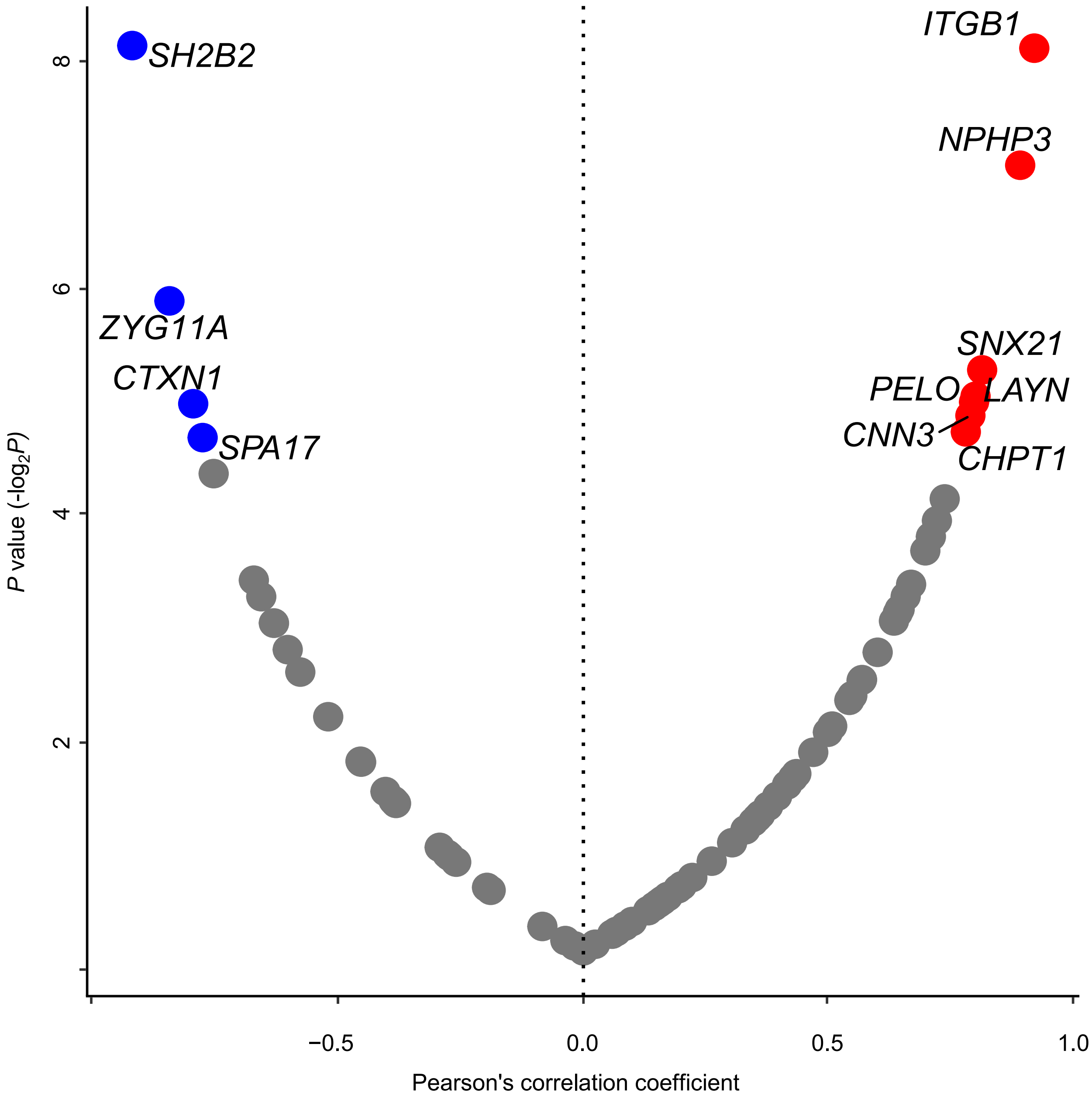

Supplementary Figure S7

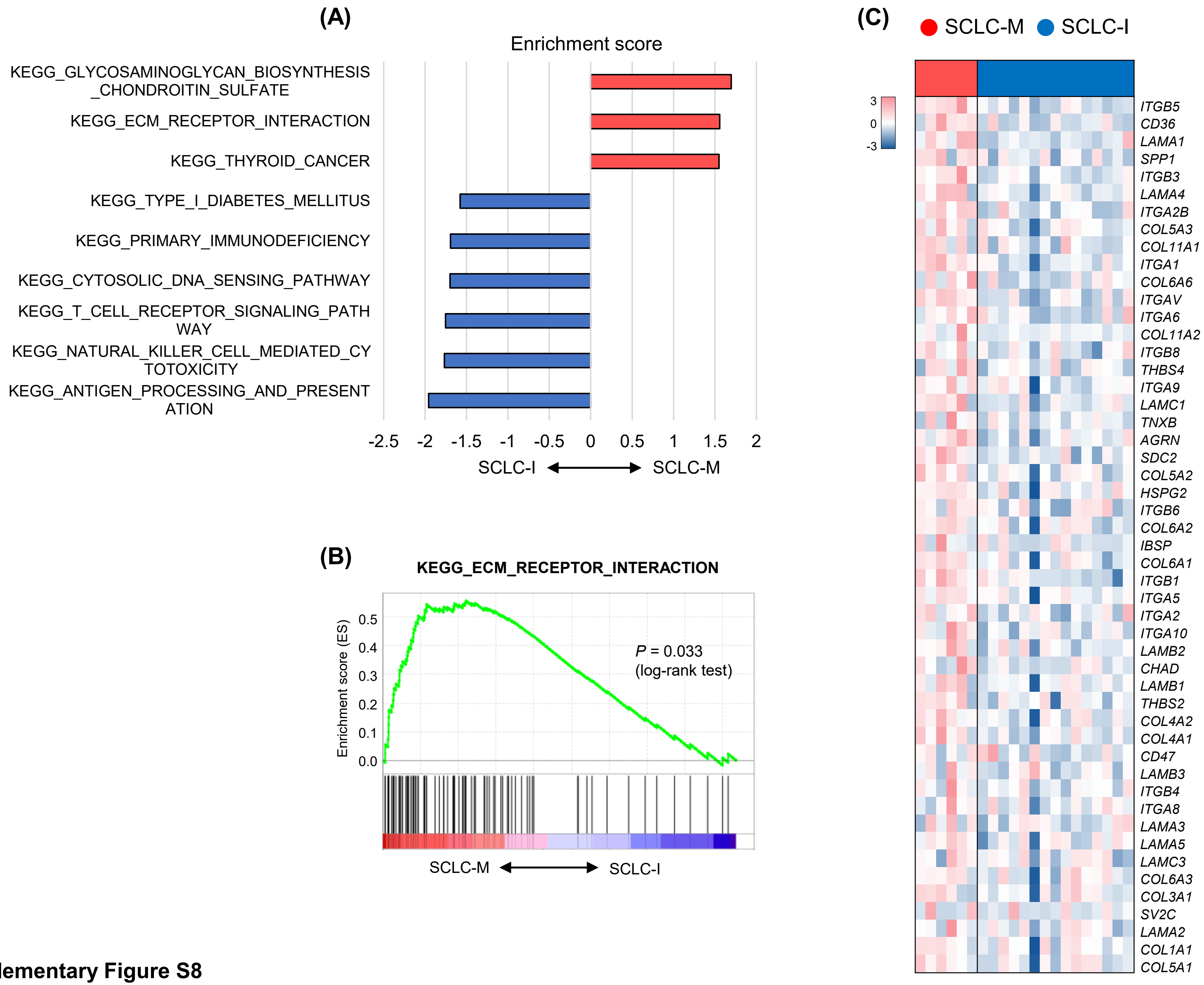

Supplementary Figure S8
